# Microglial activation and tau burden predict cognitive decline in Alzheimer’s disease

**DOI:** 10.1093/brain/awaa088

**Published:** 2020-05-07

**Authors:** Maura Malpetti, Rogier A Kievit, Luca Passamonti, P Simon Jones, Kamen A Tsvetanov, Timothy Rittman, Elijah Mak, Nicolas Nicastro, W Richard Bevan-Jones, Li Su, Young T Hong, Tim D Fryer, Franklin I Aigbirhio, John T O’Brien, James B Rowe

**Affiliations:** a1 Department of Clinical Neurosciences, University of Cambridge, Cambridge, UK; a2 MRC Cognition and Brain Sciences Unit, University of Cambridge, Cambridge, UK; a3 Institute of Molecular Bioimaging and Physiology, National Research Council, Milano, Italy; a4 Department of Psychiatry, University of Cambridge, Cambridge, UK; a5 Department of Clinical Neurosciences, Geneva University Hospitals, Switzerland; a6 Cambridge University Hospitals NHS Trust, Cambridge, UK

**Keywords:** Alzheimer’s disease, neuroinflammation, tau pathology, PET imaging, cognitive decline

## Abstract

Tau pathology, neuroinflammation, and neurodegeneration are key aspects of Alzheimer’s disease. Understanding whether these features predict cognitive decline, alone or in combination, is crucial to develop new prognostic measures and enhanced stratification for clinical trials. Here, we studied how baseline assessments of *in vivo* tau pathology (measured by ^18^F-AV-1451 PET), neuroinflammation (measured by ^11^C-PK11195 PET) and brain atrophy (derived from structural MRI) predicted longitudinal cognitive changes in patients with Alzheimer’s disease pathology. Twenty-six patients (*n = *12 with clinically probable Alzheimer’s dementia and *n = *14 with amyloid-positive mild cognitive impairment) and 29 healthy control subjects underwent baseline assessment with ^18^F-AV-1451 PET, ^11^C-PK11195 PET, and structural MRI. Cognition was examined annually over the subsequent 3 years using the revised Addenbrooke’s Cognitive Examination. Regional grey matter volumes, and regional binding of ^18^F-AV-1451 and ^11^C-PK11195 were derived from 15 temporo-parietal regions characteristically affected by Alzheimer’s disease pathology. A principal component analysis was used on each imaging modality separately, to identify the main spatial distributions of pathology. A latent growth curve model was applied across the whole sample on longitudinal cognitive scores to estimate the rate of annual decline in each participant. We regressed the individuals’ estimated rate of cognitive decline on the neuroimaging components and examined univariable predictive models with single-modality predictors, and a multi-modality predictive model, to identify the independent and combined prognostic value of the different neuroimaging markers. Principal component analysis identified a single component for the grey matter atrophy, while two components were found for each PET ligand: one weighted to the anterior temporal lobe, and another weighted to posterior temporo-parietal regions. Across the whole-sample, the single-modality models indicated significant correlations between the rate of cognitive decline and the first component of each imaging modality. In patients, both stepwise backward elimination and Bayesian model selection revealed an optimal predictive model that included both components of ^18^F-AV-1451 and the first (i.e. anterior temporal) component for ^11^C-PK11195. However, the MRI-derived atrophy component and demographic variables were excluded from the optimal predictive model of cognitive decline. We conclude that temporo-parietal tau pathology and anterior temporal neuroinflammation predict cognitive decline in patients with symptomatic Alzheimer’s disease pathology. This indicates the added value of PET biomarkers in predicting cognitive decline in Alzheimer’s disease, over and above MRI measures of brain atrophy and demographic data. Our findings also support the strategy for targeting tau and neuroinflammation in disease-modifying therapy against Alzheimer’s disease.

## Teaser

Malpetti *et al.* compare the prognostic value of neuroimaging markers for tau pathology, neuroinflammation and neuronal loss in Alzheimer’s disease. Higher temporoparietal tau burden and neuroinflammation at baseline predict faster cognitive decline over 3 years, and are more prognostic than baseline MRI measures of atrophy.

## Introduction

The pathological hallmarks of Alzheimer’s disease are tau neurofibrillary tangles and amyloid-β plaques, but neuroinflammation has also emerged as a key process in Alzheimer’s disease and other neurodegenerative disorders (Pasqualetti *et al.*, 2015; Ransohoff, 2016; Schain and Kreisl, 2017). The differential role of these pathologies in predicting clinical progression of Alzheimer’s disease remains to be ascertained. This represents a critical step to develop new prognostic markers and test the effect of novel disease-modifying therapies that target different pathologies in Alzheimer’s disease.

The aggregation of misfolded tau protein is associated with synaptic dysfunction and neuronal loss, and correlates with clinical severity in the Alzheimer’s disease clinical spectrum (Nelson *et al.*, 2012; Spires-Jones and Hyman, 2014). A significant presence of amyloid-β plaques is also indicative of likely cognitive decline in mid- and later-life, although the association of both neurodegeneration and cognitive impairment has been found stronger with the distribution and burden of neurofibrillary tangles than it is for neuritic plaques (Nelson *et al.*, 2012; Spires-Jones and Hyman, 2014). Microglia activation and neuroinflammation represent a third key determinant in the aetiopathogenesis of Alzheimer’s disease and in its progression (Heneka *et al.*, 2015; Mhatre *et al.*, 2015; Calsolaro and Edison, 2016), independently or synergistically with tau and amyloid pathology.

Each of these processes can now be quantified and localized *in vivo* using brain imaging, such as PET imaging with radioligands targeting tau pathology, amyloid burden, and microglial activation (see Chandra *et al.*, 2019 for a review). The PET ligand ^18^F-AV-1451 is sensitive to cortical tau accumulation in Alzheimer’s disease, and has high affinity for the characteristic paired helical tau filaments (Xia *et al.*, 2013; Marquié *et al.*, 2015; Lowe *et al.*, 2016). ^18^F-AV-1451 PET studies have shown marked tau accumulation in the entorhinal cortex in patients with mild cognitive impairment (MCI) that extends to temporo-parietal regions in Alzheimer’s disease (Hall *et al.*, 2017). ^18^F-AV-1451 bindings also correlates with Braak staging of neurofibrillary tau (Schöll *et al.*, 2016; Schwarz *et al.*, 2016), and post-mortem patterns of Alzheimer’s disease pathology (Sander *et al.*, 2016; Lowe *et al.*, 2020; Smith *et al.*, 2019). This is also in keeping with evidence that tau deposition is evident as a continuum from normal ageing through MCI to Alzheimer’s dementia (Schöll *et al.*, 2019), and correlates with cognitive impairment (Brier *et al.*, 2016; Cho *et al.*, 2016*b*; Johnson *et al.*, 2016; Ossenkoppele *et al.*, 2016; Pontecorvo *et al.*, 2017). In addition, PET imaging supported the previous evidence of a stronger association of cognitive deficits with tau burden than with amyloid-β (Brier *et al.*, 2016; Johnson *et al.*, 2016).

The PET ligand ^11^C-PK11195 is a well-established marker for microglial activation via its binding to the 18-kDa translocator protein (TSPO), a mitochondrial membrane protein that is overexpressed in activated microglia (Scarf and Kassiou, 2011). Results with this ligand in Alzheimer’s disease have been variable (for a review see Chandra *et al.*, 2019), but this may be due to small sample sizes and inconsistent methods between previous *in vivo* studies. ^11^C-PK11195 has shown high binding in temporo-parietal regions and cingulate cortex in patients with Alzheimer’s disease (Stefaniak and O’Brien, 2015), while neuroinflammation in these regions is inversely associated with cognitive performance in MCI and Alzheimer’s dementia (Edison *et al.*, 2008; Okello *et al.*, 2009*a*; Fan *et al.*, 2015*a*; Passamonti *et al.*, 2018, 2019). However, inflammation does not correlate well with amyloid burden (Yokokura *et al.*, 2011), suggesting an independent role of microglia activation in leading to cognitive deficits.

There are extensive data on atrophy in Alzheimer’s disease, measured in terms of volume loss *in vivo* by MRI, at MCI and dementia stages of progressive Alzheimer’s disease pathology. For example, MRI measures of medial temporal lobe volumes correlate with disease severity, and are predictive of future conversion from MCI to Alzheimer’s disease (Frisoni *et al.*, 2010; Leung *et al.*, 2013; Jack *et al.*, 2015). However, cell loss and atrophy are relatively late features in a cascade of pathology, and it is not clear how MRI compares with measures of molecular pathology as a prognostic marker, especially in view of marked age-related structural changes (Raz *et al.*, 2005; Walhovd *et al.*, 2011).

In this study, we test the ability of baseline *in vivo* measures of tau pathology, microglia activation, and brain atrophy to predict the rate of cognitive decline in patients with Alzheimer’s disease pathology, ranging from MCI (with biomarker evidence of amyloid pathology) to clinically probable Alzheimer’s disease (with dementia). Our hypothesis was that the PET biomarkers of tau pathology and neuroinflammation are strong predictors of cognitive impairment and decline, and that whereas MRI may be predictive in isolation, the prognostic information of MRI is better captured by direct PET measures of molecular pathology (Bejanin *et al.*, 2017; Mattsson *et al.*, 2019). This hypothesis builds on evidence that tau burden relates to age-related cognitive decline (Schöll *et al.*, 2016; Aschenbrenner *et al.*, 2018; Maass *et al.*, 2018), and progression of dementia over 6 to 18 months in patients with Alzheimer’s disease (Koychev *et al.*, 2017; Pontecorvo *et al.*, 2019). In contrast to past studies that assessed the relationship between longitudinal PET markers and clinical changes in Alzheimer’s disease (Fan *et al.*, 2015*b*, 2017, Chiotis *et al.*, 2018; Jack *et al.*, 2018; Southekal *et al.*, 2018; Cho *et al.*, 2019), we studied how a multi-modal and cross-sectional assessment of distinct pathologies is able to predict longitudinal decline in Alzheimer’s disease, examining the individual or combined prognostic contribution of tau pathology, neuroinflammation, and brain atrophy in predicting cognitive decline.

The better characterization of the factors predicting decline in Alzheimer’s disease will help to develop enhanced prognostic and outcome measures for clinical trials targeting more than one pathology. Although previous findings support the use of MRI and PET imaging in the diagnosis and monitoring of disease progression, the prognostic value of these *in vivo* measures and their combined effect in predicting clinical decline in Alzheimer’s disease remains undetermined. Previous studies that have evaluated the predictive values of neuroimaging markers in Alzheimer’s disease have typically assessed different neuroimaging modalities in isolation rather than exploiting the mechanistic and prognostic values that are offered by multi-modal neuroimaging. We therefore assessed the independent and combined predictive effects of baseline neuroimaging biomarkers for tau pathology (^18^F-AV-1451 PET), neuroinflammation (^11^C-PK11195 PET) and brain atrophy (structural MRI) on longitudinal cognitive changes over a period of 3 years in the clinical spectrum of Alzheimer’s disease. Given the published evidence of dominant involvement of temporal and parietal brain regions in early neurodegeneration, tau pathology, and neuroinflammation in Alzheimer’s disease (Garibotto *et al.*, 2017; Jagust, 2018; Whitwell, 2018), we decided *a priori* to focus our analyses on these regions. Pathology may occur in frontal and occipital regions, but for typical amnestic phenotypes, we considered this to be of secondary importance. In the temporo- parietal regions there is a hierarchical evolution in tau pathology and atrophy from MCI to Alzheimer’s dementia, recapitulating neuropathological staging and correlating with clinical severity (for a review see Jagust, 2018).

We predicted: (i) a significant association between baseline measures of each neuroimaging technique and longitudinal decline in cognition; (ii) partially independent and additive effects of MRI and PET measures on cognitive decline, assessed with all modalities together in a single multivariate model; and (iii) that the molecular markers of baseline tau and neuroinflammation PET would be more informative than structural MRI on longitudinal cognitive deterioration in Alzheimer’s disease.

## Material and methods

### Participants

We recruited 26 patients: 12 with a clinical diagnosis of probable Alzheimer’s dementia and 14 with amnestic MCI and a positive amyloid PET scan as biomarker of Alzheimer’s disease (Klunk *et al.*, 2004). Probable Alzheimer’s dementia was diagnosed according to the National Institute on Aging-Alzheimer’s Association guidelines (McKhann *et al.*, 2011) and confirmed in all patients during follow-up. Given the long-term and intensive nature of the longitudinal project, all patients at baseline had >12/30 on the Mini-Mental State Examination (MMSE) to be eligible to participate in the study. MCI patients had MMSE score >24/30, and memory impairment not ascribable another diagnosis (Albert *et al.*, 2011). We also included 29 healthy controls with MMSE >26/30, absence of memory symptoms, no signs of dementia, or any other significant medical illnesses.

All gave informed consent according to the Declaration of Helsinki. The NIMROD protocol [Neuroimaging of Inflammation in Memory and Related Other Disorders (Bevan-Jones *et al.*, 2017)] was approved by the NIHR National Research Ethic Service Committee and East of England (Cambridge Central).

During the first visit, demographic information and medical history were collected. All participants underwent a baseline neuropsychological assessment, followed by an MRI scan and one, two or three PET scans depending on the group. The clinical examination and neuropsychological battery were repeated annually for three follow-up visits (for details see Bevan-Jones *et al.*, 2017). The revised Addenbrooke’s Cognitive Examination (ACE-R) (Mioshi *et al.*, 2006) was used to assess the cognitive performance at each visit. All patients diagnosed with Alzheimer’s dementia deteriorated significantly in the follow-up clinical visits compared to the study baseline. Six of 14 patients with MCI were clinically diagnosed as converting to Alzheimer’s disease and/or presented MMSE ≤24/30 by the end of the study (3 years), and three further patients subsequently.

### Imaging data acquisition and preprocessing

All subjects underwent 3 T MRI performed on a Siemens Magnetom Tim Trio or Verio scanner (Siemens). A T_1_-weighted MPRAGE image was acquired for each participant (repetition time = 2300 ms, echo time = 2.98 ms, field of view = 240 × 256 mm^2^, 176 slices of 1-mm thickness, flip angle = 9°).

MCI and Alzheimer’s dementia subjects had both dynamic ^18^F-AV-1451 PET and ^11^C-PK11195 PET, while, to minimize radiation exposure in healthy individuals, control subjects were divided into two groups: 14 underwent ^18^F-AV-1451 PET, while another 15 underwent ^11^C-PK11195 PET. PET scanning was undertaken on a GE Advance PET scanner (GE Healthcare) and a GE Discovery 690 PET/CT (Supplementary Table 1). Patients with MCI also underwent 40–70 min post-injection ^11^C-PiB PET to quantify the density of fibrillar amyloid-β deposits for classification of amyloid-β status. The emission protocols were: 90 min dynamic imaging following a 370 MBq ^18^F-AV-1451 injection; 75 min of dynamic imaging starting concurrently with a 500 MBq ^11^C-PK11195 injection; and 550 MBq ^11^C-PiB injection followed by imaging from 40–70 min post-injection (for details see Passamonti *et al.*, 2017, 2018). All images were reconstructed with PROMIS 3D filtered back-projection (Kinahan and Rogers, 1989), with the Colsher filter apodized with a transaxial Hann filter cut-off at the Nyquist frequency. Corrections for dead time, randoms, normalization, scatter, attenuation, and sensitivity were included in the image reconstruction process. ^11^C-PiB scans were classified as positive if the average standardized uptake value ratio (SUVR) across the cortex using a cerebellar grey matter reference region was >1.5 (Villemagne *et al.*, 2013). This threshold was chosen to minimize false positives (Jack *et al.*, 2008; Villemagne *et al.*, 2011). Only MCI patients with positive amyloid-β status were included in this study, and were combined with patients with Alzheimer’s dementia as these two groups are thought to represent a continuum of the same clinical and pathological spectrum (Okello *et al.*, 2009*b*).

Structural imaging data were processed in SPM12. The T_1_-weighted images were segmented into grey matter, white matter and CSF and used to determine regional grey matter, white matter and CSF volumes, and to calculate brain volume (grey + white matter) and total intracranial volume (TIV = grey matter + white matter + CSF) in each participant. The grey and white matter segments from 33 subjects were used to create an unbiased template (11 controls, 11 patients with MCI and 11 patients with Alzheimer’s dementia, matched for age and sex across the groups) using the DARTEL pipeline in SPM12. The images from the remaining 22 participants were warped to the template to bring all participants into the same space. Segmented images were then warped to MNI space. The images were matched to the Hammers atlas [(Hammers *et al.*, 2003; Gousias *et al.*, 2008) modified to include brainstem parcellation and the cerebellar dentate nucleus in MNI152 2009c space] to perform a region of interest analysis. The atlas comprised 83 cortical regions. The group template was warped to the ICBM MNI152 2009c template using the ‘Population to ICBM’ function, applied to the Hammers atlas in DARTEL template space, followed by linear transformation to MNI space. These steps place the regions of interest in the same space as the individual normalized MRI images. Individual regional grey matter volumes were then extracted using the ‘spm_summarise’ function.

For each subject, the aligned PET image series for each scan was rigidly co-registered to the T_1_-weighted MRI image. Prior to kinetic modelling, regional PET data were corrected for CSF contamination by dividing by the mean region grey plus white matter fraction determined from SPM tissue probability maps smoothed to PET spatial resolution. For ^11^C-PK11195, supervised cluster analysis was used to determine the reference tissue time-activity curve and non-displaceable binding potential (BP_ND_) was calculated in each region of interest using a simplified reference tissue model that includes correction for vascular binding (Yaqub *et al.*, 2012). For ^18^F-AV-1451, BP_ND_ was assessed in each region of interest with the simplified reference tissue model (Gunn *et al.*, 1997) using superior cerebellar cortex grey matter as the reference region. For more details about the data preprocessing steps, see Passamonti *et al.* (2017, 2018).

The number of regions was reduced from 83 to 15 *a priori* regions of interest by (i) combining left and right regional values in bilateral regions (*cf.*Passamonti *et al.*, 2017, 2018); and (ii) focusing on 15 bilateral temporo-parietal regions, related to Alzheimer’s disease pathology (Supplementary Table 2). Regional grey matter volumes were corrected for total intracranial volume. For both ^11^C-PK11195 and ^18^F-AV-1451, a volume-weighted mean of left and right regional BP_ND_ values was calculated for each bilateral region of interest.

### Statistical analyses

#### Descriptive statistics

Continuous variables (age, education, ACE-R) were compared between groups with an independent-samples *t*-test, and categorical variables (sex) with the chi-square test. The effect size of each *t*-test comparison was computed to quantify differences between the two groups (Cohen’s *d *>* *0.8, valuable difference).

#### Principal components analysis

The standardized values determined for the 15 bilateral regions of interest from each imaging dataset were included in three principal component analyses (PCAs), run separately for grey matter volumes, ^11^C-PK11195 and ^18^F-AV-1451 BP_ND_ values. This reduces dimensionality and the problem of multiple comparisons, identifying a limited number of components that best explain the data variance. We applied an orthogonal varimax rotation to maximize interpretability and specificity of the resulting components. We retained components with eigenvalues >1. To test whether correction for CSF affected the PCA results, we applied the same analyses on ^18^F-AV-1451 PET and ^11^C-PK11195 regional data not corrected for CSF partial volume.

The individual component scores were corrected for the time interval in months between the baseline cognitive assessment and each scan. Median (mean and standard deviation, SD) of the time interval between the baseline cognitive assessment and the imaging scans were: 1.0 (1.75 ± 2.50) months for MRI, 7.5 (7.18 ± 5.68) months for ^18^F-AV-1451 PET and 2 (6.12 ± 9.07) months for ^11^C-PK11195 PET. The residuals extracted for each component were included in a multiple regression on cognitive decline as independent variables.

#### LGCM for cognitive data

ACE-R scores at follow-up were annualized to the nearest whole year, using the absolute difference in scores between the baseline and the following visits, divided by the time interval in days between tests and multiplied by 365 (1 year), 730 (2 years) or 1095 (3 years). A latent growth curve model (LGCM) was fitted on longitudinal annualized ACE-R scores across all subjects (*n = *55), to obtain (i) the intercept; (ii) the slope, quantifying the rate of change and its form (i.e. linear or non-linear); and (iii) the relation between intercept and slope. A linear slope for the longitudinal ACE-R scores was estimated, and used in further analyses. Addition of a non-linear (quadratic) term to the model did not improve the estimation of slope (Supplementary material). The estimated parameters are based on the individuals’ trajectory, indicating average change and individual difference. Covariates can be added to the model to assess their associations with both intercept and slope. Three time points and 5–10 cases per parameter are required for a standard LGCM (Bentler and Chou, 1987; Newsom, 2015). LGCM was implemented in Lavaan software (Rosseel, 2012) using full information maximum likelihood estimation with robust standard errors for missingness and non-normality. We considered four indices of good model fit (Schermelleh-Engel *et al.*, 2003): (i) the chi-square test with the *P*-value (good fit: >0.05); (ii) the root-mean-square error of approximation (RMSEA, acceptable fit: <0.08, good fit: <0.05); (iii) the comparative fit index (CFI, acceptable fit: 0.95–0.97, good fit: >0.97); and (iv) the standardized root mean-square residual (SRMR, acceptable fit: 0.05–0.10, good fit: <0.05). From the model fitting, variables ‘intercept’ and ‘slope’ were created extracting the individual estimated values for each subject in the model. *T*-tests and ANOVA tested for group differences in initial cognitive performance and annual change.

#### One-step prediction procedure: LGCMs with predictors

Across all subjects, we tested the predictive value of each imaging method on cognitive decline, applying five LGCMs with each scan-specific component’s values (corrected for months from the baseline) as predictor of cognitive intercept and slope. Models were tested separately for MRI (*n = *55), ^11^C-PK11195 PET (*n = *41) and ^18^F-AV-1451 PET (*n = *40). Then, in patients (*n = *26), the individual scores of all five imaging components were included as predictors in the LGCM on longitudinal ACE-R, estimating the combined predictive effect of imaging modalities.

The one step procedure is a simple approach to our research questions, but brings estimation challenges with a modest sample size. Therefore, we next applied a two-step prediction procedure: (i) extracting individual slope values from the initial LGCM for cognitive data across all the population; and (ii) including these values as dependent variables in linear regression models with brain imaging components as predictors. We present both frequentist and Bayesian analyses to ensure inferential robustness and allowing us to quantify evidence in favour of the null hypothesis (of no predictive value).

#### Two-step frequentist prediction: linear regression models on LGCM parameters

First, across all subjects, the residual values of each scan-specific PCA component (corrected for months from the baseline) were included as single predictors in separated univariable linear regression models with the individual slope values extracted from the initial LGCM as dependent variable. The significance level was set at *P < *0.01 corrected for multiple comparisons (Bonferroni correction *α*  =  0.05/5). Next, the individual scores of the imaging methods’ components were included as independent variables in a multivariable regression analysis on patients alone (*n *=* *26), who underwent all three imaging scans. This model was fit to examine the individual, as well as combined, ability to explain variance in cognitive decline using brain marker components as well as age, education, and sex as independent variables. The model used stepwise backward selection (entry criterion *α*  =  0.05 and elimination criterion *α*  =  0.1; see the Supplementary material for comparable models applied with the estimated intercept as dependent variable). A complementary *post hoc* ‘exploratory’ linear regression analysis included the interaction term between PET independent variables, to test whether their interaction was predictive of cognitive decline. In supplementary analyses, we applied a ‘reduced’ multivariable linear regression analysis with slope as dependent variable and only the first component of each imaging method as predictors to test that the different number of components between MRI and PET did not affect the estimation. These supplementary analyses were performed with and without the interaction terms between tau and inflammation measures. Given the challenges of stepwise model selection, and the limitations of sample size to utilize more advanced methods (e.g. regularized model fitting), we ran the analysis using Bayesian methodology, to ensure inferential robustness of our findings and confidence in the null results.

### Two-step Bayesian prediction: linear regression models on LGCM estimated parameters

We applied a Bayesian multiple regression analysis with brain components and demographic variables as predictors, and the estimated slope values as dependent variable. This approach was used to test whether there was evidence for the absence of independent variables’ effect for those components excluded from the final models (as opposed to frequentist type II error). In the model comparisons, adopting a uniform prior over the models, we considered as final model the one with the highest Bayes Factor compared to the null model (BF_10_). Then, we used a reduced Bayesian linear regression, mirroring the reduced model applied with the frequentist approach, which included only the first component of each imaging method as predictors of slope.

#### One- and two-step prediction procedures

See Supplementary Fig. 1 for a schematic representation of statistical analyses with one-step and two-step prediction procedures. All PCAs and regression models were performed in SPSS Statistics version 25 (IBM); all Bayesian analysis in JASP version 0.10.2 (JASP team) and all LCGMs used R version 3.6.1 (R Core Team).

### Data availability

Anonymized data may be shared by request to the senior author from a qualified investigator for non-commercial use (sharing of some data is subject to restrictions according to participant consent and data protection legislation).

## Results

### Descriptive statistics

**Figure 1:**
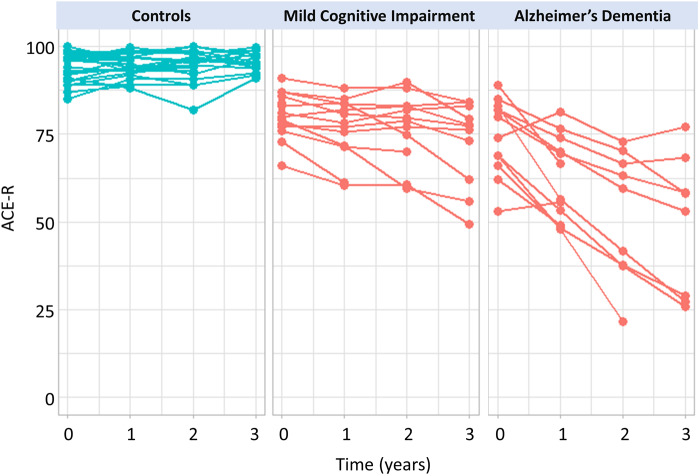
**Longitudinal cognitive changes in patients and controls, as measured by the ACE-R.** Points represent annualized ACE-R scores at baseline, and 1-year, 2-year and 3-year follow-ups for each subject in control (blue) and patient (red) groups.

Significant differences between patient and control groups were found for education [*t*(48.4) = 2.4, *P = *0.02, *d *=* *0.64] and ACE-R scores [*t*(33.3) = 8.6, *P < *0.001, *d *=* *2.37]. There were no significant group differences in age [*t*(53) = −1.7, *P = *0.09, *d* = −0.47] and sex [χ^2^(1) = 0.17, *P = *0.68] (Table 1). Individual ACE-R scores at baseline and at each follow-up are shown in Fig. 1. See Supplementary Table 3 for demographics in patient and control subgroups.

**Table 1:** Demographic and clinical characteristics for the patient and control groups

	MCI+/AD patients	Healthy controls	Group difference
*n*	26	29	
Sex, female/male	12/14	15/14	χ^2^(1) = 0.17, *P =* 0.68
Age, years, mean ± SD	72.1 ± 8.7	68.3 ± 7.2	*t*(53) = −1.7, *P =* 0.09, *d* = −0.47
Education, years, mean ± SD	13.1 ± 3.2	14.9 ± 2.6	*t*(48.4) = 2.4, *P =* 0.02, *d* = 0.64
ACE-R Baseline, mean ± SD	77.8 ± 9.1	94.4 ± 4.0	*t*(33.3) = 8.6, *P* < 0.001, *d* = 2.37 *
Disease duration, years, mean ± SD	3.6 ± 2.1	–	–

* Significant difference between patients and controls (*P*-value < 0.05) with effect size *d *>* *0.8 for *t*-test.

AD = Alzheimer’s disease.

### Principal component analysis of grey matter volumes, ^18^F-AV-1451 BP_ND_ and ^11^C-PK11195 BP_ND_

**Figure 2 Principal components of the multimodal imaging.**
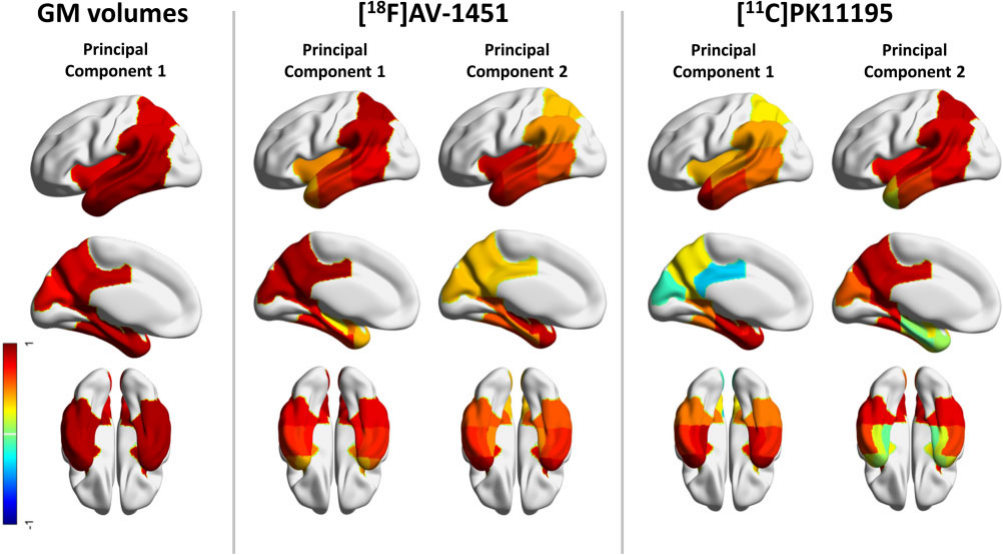
Regional weights of the structural MRI component (*left*), and rotated regional weights of ^18^F-AV-1451 components (*middle*) and the ^11^C-PK11195 components (*right*). Components were identified applying three independent principal component analyses on 15 temporo-parietal regions. For structural MRI, regional grey matter (GM) volumes were included in the analysis, while for each PET tracer, the binding potential values in those regions were considered, separately for each modality. The colours represent the region-specific weights (range: from −1 to 1) on each component (Supplementary Table 4).

For grey matter volumes, the PCA on the preselected 15 Alzheimer’s disease-specific cortical regions identified only one component that encompassed all the temporo-parietal regions and explained 74% of the variance (Fig. 2, left). Two principal components were detected for ^18^F-AV-1451 BP_ND_ data, explaining 91% of the total variance (83% first component; 8% second component). The first component was loaded onto the posterior temporal and parietal regions, while the second component was weighted to the anterior temporal lobe, amygdala, insula and hippocampus (Fig. 2, middle). For ^11^C-PK11195 BP_ND_ data, two principal components were identified, and these explained together the 76% of data variance (56% for the first component; 20% for the second component). The first component involved anterior and medial temporal lobe, while the second component was mainly loaded onto the posterior temporo-parietal regions and insula (Fig. 2, right). The loadings are shown in Supplementary Table 4. Using PET data without CSF correction yielded qualitatively similar results.

Simple correlations between MRI and PET component scores across subjects were significant for the MRI component versus the first ^11^C-PK11195 component (R = −0.459, *P = *0.003, significant after Bonferroni correction), but not the second ^11^C-PK11195 component (R = −0.154, *P = *0.337). The MRI component was weakly associated with the first (R = −0.319, *P = *0.045, uncorrected) and the second (R = −0.329, *P = *0.038, uncorrected) ^18^F-AV-1451 components. In patients, correlations between ^18^F-AV-1451 and ^11^C-PK11195 components were not significant, even uncorrected.

### Annual rate of cognitive decline

**Figure 3:**
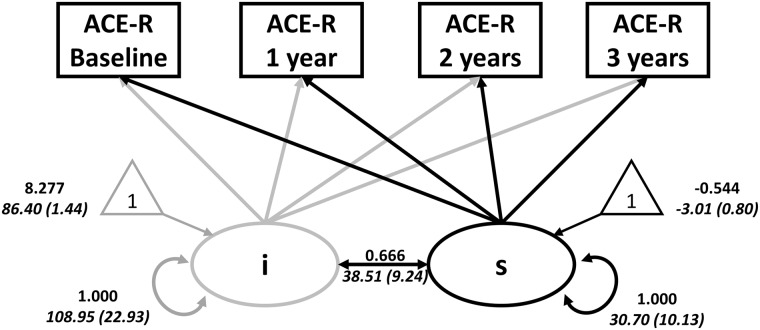
**LGCM to test the initial values (intercept, ‘i’) and longitudinal changes (slope, ‘s’) in scores of the ACE-R across all sample.** Circles indicate latent variables, rectangles indicate observed variables, and triangles denote intercepts (1 = population means on the parameters). Thick single-headed arrows indicate regressions while thick double-headed arrows indicate variance and covariance (grey for intercept and black for slope). Values in Roman are standardized parameter estimates, and values in italics are unstandardized parameter estimates (with standard errors in parentheses). The annual rate of change was positively associated with performance at baseline (lower initial cognitive scores were associated with a higher annual rate of cognitive changes).

The linear LGCM of longitudinal ACE-R scores fitted the data adequately [χ^2^(8) = 10.93, *P = *0.206; RMSEA = 0.09 (0.00–0.21), CFI = 0.99, SRMR = 0.04]. Three of four model fit indices were ‘acceptable’ or ‘good’ by Schermelleh-Engel *et al.* (2003) guidelines, although the RMSEA (>0.08) was not. To exclude a large single source of misfit, we inspected the standardized residual matrix, and confirmed no single standardized residual greater than r = 0.099. We therefore considered the overall model fit sufficient. The mean of the intercept was 86.40 [standard error (SE) = 1.44, *z*-value = 60.02, fully standardized estimate (Std Est) = 8.28, *P < *0.001] and average cognition declined over time [slope, estimate (est) = −3.01, SE = 0.80, *z*-value = −3.75, Std Est = −0.54, *P < *0.001]. The intercept significantly covaried with the slope [est = 38.51, SE = 9.24, *z*-value = 4.17, Std Est (correlation) = 0.67, *P < *0.001], such that individuals with higher (better) baseline performance showed less steep decline. As expected, patients significantly differed from controls in their intercept [*t*(31.8) = 9.39, *P < *0.001] and slope [*t*(25.9) = 6.42, *P < *0.001] indicating a faster and more severe cognitive decline (Fig. 3). Across three groups, ANOVA confirmed group differences in the intercept [*F*(2) = 63.44, *P < *0.001; mean (SD) for: controls = 94.18 (3.27); MCI-positive patients = 81.25 (6.17); Alzheimer’s patients = 73.60 (8.96)] and slope [*F*(2) = 53.74, *P < *0.001; mean (SD) for: controls = 0.40 (0.82); MCI-positive patients = −3.56 (3.08); Alzheimer’s patients = −10.62 (5.71)], with *post hoc* confirmation of differences between each pair of groups (all *P < *0.005).

### One-step prediction LGCM with predictors

The LGCM including MRI fitted the data adequately [χ^2^(10) = 18.33, *P*-value = 0.05, RMSEA = 0.13 (0.01–0.23), CFI = 0.98, SRMR = 0.03]. Inspecting the standardized residual matrices, none were greater than *r = *0.105. Individual differences in the summary brain measure were strongly and positively associated with both slope (path Std Est = 0.58, *P < *0.001) and intercept (path Std Est = 0.67, *P < *0.001). This suggested that individuals with greater grey matter volumes showed better baseline performance, and slower longitudinal decline, than those with smaller volumes.

The LGCM of the posterior ^18^F-AV-1451 component fitted the data adequately [χ^2^(10) = 16.30, *P*-value = 0.09, RMSEA = 0.12 (0.00–0.22), CFI = 0.98, SRMR = 0.03], and no single standardized residual was greater than *r = *0.101. Here too, both the slope (path Std Est = −0.62, *P = *0.001) and intercept (path Std Est = −0.53, *P < *0.001) were strongly governed by individual differences in the first component. In contrast, in the model with only the anterior ^18^F-AV-1451 component [χ^2^(10) = 21.75, *P*-value = 0.01, RMSEA = 0.17 (0.07–0.27), CFI = 0.96, SRMR = 0.05], there was no association between the scores on the neural component and either the intercept (path Std Est = −0.12, *P = *0.431) or the slope (path Std Est = −0.39, *P = *0.057). In this model, no single standardized residual was greater than *r = *0.148.

Finally, the LGCM with the anterior ^11^C-PK11195 component fitted the adequately [χ^2^(10) = 16.32, *P*-value = 0.09, RMSEA = 0.13 (0.00–0.23), CFI = 0.97, SRMR = 0.02], and no single standardized residual was greater than *r = *0.056. Individual differences in the ^11^C-PK11195 component governed both slope (Std Est = −0.51, *P = *0.002) and intercept (Std Est = −0.43, *P < *0.001) correlated with Component 1. In the model with the posterior ^11^C-PK11195 component as regressor, [χ^2^(10) = 9.33, *P*-value = 0.50, RMSEA = 0.00 (0.00–0.17), CFI = 1.00, SRMR = 0.03], the slope resulted significantly correlated with the component (Std Est = −0.45, *P = *0.009), but not the intercept (Std Est = −0.010, *P = *0.951). No single standardized residual was greater than *r = *0.108.

In patients, an LGCM including the components of all three imaging methods did not fit the data well [χ^2^(18) = 34.76, *P*-value = 0.01, RMSEA = 0.17 (0.08–0.26), CFI = 0.92, SRMR = 0.04], but no single standardized residual was greater than *r = *0.119. With this caveat, cognitive decline (slope) was predicted by baseline posterior ^18^F-AV-1451 (path Std Est = −0.49, *P = *0.025) and anterior ^11^C-PK11195 (path Std Est = −0.40, *P = *0.017) components’ scores, but not the posterior ^11^C-PK11195, the MRI (path Std Est = 0.10, *P = *0.52) or the anterior ^18^F-AV-1451 (Std Est = −0.22, *P = *0.23) components.

### Two-step prediction: linear regression

**Figure 4:**
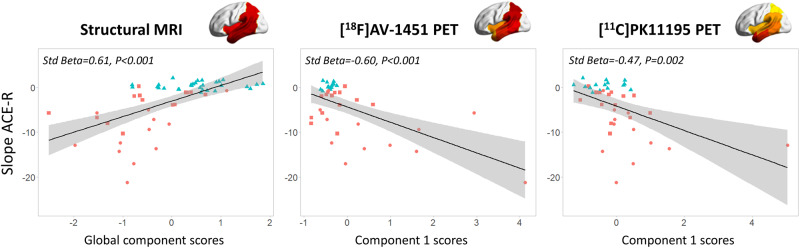
**Imaging predictors of cognitive decline.** Regression analyses with annual change in scores of the ACE-R (Slope ACE-R, *y*-axis) and individual baseline scores for each modality-specific principal component (*x*-axis): structural MRI (*left*), ^18^F-AV-1451 PET (*middle*), and ^11^C-PK11195 PET (*right*). Different colours represent different diagnostic groups: red circles = patients with Alzheimer’s disease; red squares = patients with amyloid-positive MCI; controls = blue triangles.

Across all subjects, the rate of cognitive decline (slope from LGCM) was significantly associated with: (i) the MRI weighting (Std Beta = 0.61, *P < *0.001); (ii) the posterior ^18^F-AV-1451 (Std Beta = −0.60, *P < *0.001); and (iii) anterior ^11^C-PK11195 (Std Beta = −0.47, *P = *0.002). All these results survived Bonferroni’s correction. Correlations of slope with the anterior ^18^F-AV-1451 (Std Beta = −0.36, *P = *0.022), and the posterior ^11^C-PK11195 (Std Beta = −0.39, *P = *0.012) did not survive correction for multiple comparisons (*P < *0.01). Strikingly, these parameter estimates remained effectively unchanged even when simultaneously including age, sex and education as covariates in the models (Supplementary Table 5). See Fig. 4 for a graphical representation of the significant associations between individual scores (*x*-axis) of imaging-specific principal components and slope in ACE-R scores (*y*-axis) extracted by LGCM. Model summary and coefficients for all univariable models with slope as dependent variable across the whole population are reported in Table 2. See Supplementary Table 6 for results from analysis of patients only.

**Table 2:** Results for the univariable regression models on slope across all populations

Model		Estimate	SE	Std Beta	*t*-value	*P*	Adjusted R^2^ (SE)	*F*	*P*
MRI (*n =* 55)	(Intercept)	−3.01	0.58		−5.23	0.000	0.358 (4.27)	31.18	<0.001*
	MRI component	3.48	0.63	0.61	5.58	0.000			
AV 1 (*n =* 40)	(Intercept)	−4.31	0.73	–	−5.88	0.000	0.341 (4.64)	21.22	<0.001*
	AV component 1	−3.43	0.74	−0.60	−4.61	0.000			
AV 2 (*n =* 40)	(Intercept)	−4.31	0.85	–	−5.05	0.000	0.108 (5.40)	5.72	0.022
	AV component 2	−2.08	0.87	−0.36	−2.39	0.022			
PK 1 (*n =* 41)	(Intercept)	−4.15	0.80	–	−5.19	0.000	0.204 (5.13)	11.26	0.002*
	PK component 1	−2.72	0.81	−0.47	−3.36	0.002			
PK 2 (*n =* 41)	(Intercept)	−4.15	0.84	–	−4.96	0.000	0.128 (5.36)	6.87	0.012
	PK component 2	−2.36	0.90	−0.39	−2.62	0.012			

AV = ^18^F-AV-1451; PK = ^11^C-PK11195.

*P* = uncorrected *P*-values;

* Bonferroni corrected, significance threshold *P *<* *0.01.

**Figure 5:**
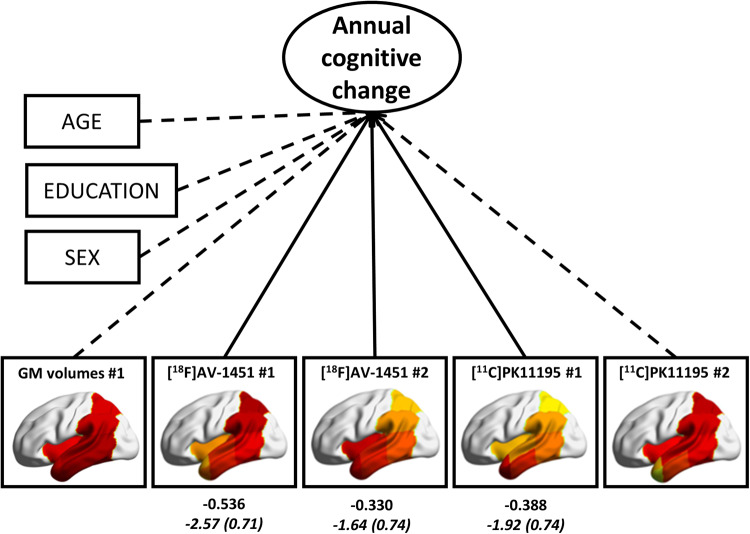
**Results of the multiple linear regression in patients, with cognitive slope (annual cognitive change) extracted by the LGCM as dependent variable, and brain components’ scores, age and education as independent variables.** Solid arrows indicate significant coefficients of brain imaging measures indicated by the stepwise backward elimination, while dashed arrows indicate variables excluded by the final model. Values in Roman are standardized estimates, and values in italics are unstandardized beta estimates (standard errors in parentheses).

In patients, the final model of multiple regression on cognitive slope (adjusted R^2^ = 0.418, SE *=* 4.18; *P = *0.001) included both ^18^F-AV-1451 components (component 1: Est = −2.57, SE *=* 0.71, *P = *0.002; component 2: Est = −1.64, SE *=* 0.74, *P = *0.038), and the anterior ^11^C-PK11195 (component 1: Est = −1.92, SE *=* 0.74, *P = *0.017) as predictors (Fig. 5 and Table 3). Of note age, education, sex, the MRI component, and the posterior ^11^C-PK11195 component were excluded from the final model.

**Table 3:** Results of the multivariable regression models on the regression slope in patients

Frequentist regression									
Final model (Stepwise backward selection)	Estimate	SE	Std Beta	*t*-value		*P*	Adjusted R^2^ (SE)	*F*	*P*
(Intercept)	−5.41	0.87		−6.19		0.000	0.418 (4.18)	8.05	0.001
AV component 1	−2.57	0.71	−0.54	−3.60		0.002			
AV component 2	−1.64	0.74	−0.33	−2.21		0.038			
PK component 1	−1.92	0.74	−0.39	−2.59		0.017			

**Bayesian regression**									

**Final model (BF-based selection)**	**Mean**	**SD**	**95% Credible interval**				**R^2^**	**BF_10_**	
			**Lower**		**Upper**				

(Intercept)	−6.82	0.82	−8.502		−5.129		0.523	46.56	
AV component 1	−2.15	0.65	−3.491		−0.802				
AV component 2	−1.37	0.68	−2.774		0.026				
PK component 1	−1.61	0.68	−3.001		−0.210				

For both frequentist (*top*) and Bayesian (*bottom*) the estimated coefficients for variables included in the final (‘best’) models are reported.

AV = ^18^F-AV-1451; BF = Bayesian factor; PK = ^11^C-PK11195.

Model summary and coefficients for both the initial model (adjusted R^2^ = 0.389, SE *=* 4.43; *P = *0.027), the full model with only brain predictors (adjusted R^2^ = 0.474, SE *=* 4.12; *P = *0.002), and the final model are reported in Supplementary Table 7. Either in the initial model with covariates or in the full model with only brain measures as predictors, the posterior ^18^F-AV-1451 component and the anterior ^11^C-PK11195 component showed the highest estimated coefficients (Supplementary Table 7). The interaction between the imaging components in the final model was not significant (*P *>* *0.05 uncorrected). In addition, the reduced multiple regression analysis, with the first component of each imaging method only, included the ^18^F-AV-1451 component (Est = −2.42, SE *=* 0.77, *P = *0.004) and the ^11^C-PK11195 component (Est = −1.71, SE *=* 0.80, *P = *0.042) in the final model (adjusted R^2^ = 0.366, SE *=* 4.52; *P = *0.002), while the MRI component was discarded. Again, the interaction between the imaging components was not significant (*P *>* *0.05 uncorrected).

### Two-step Bayesian prediction

With all brain components and demographic variables as candidate predictors of cognitive decline, model comparison using Bayes factors indicated that the best model included both ^18^F-AV-1451 components [component 1: mean (SD) = −2.15 (0.65); component 2: mean (SD) = −1.37 (0.68)], and the anterior ^11^C-PK11195 component [mean (SD) = −1.61 (0.68)] as predictors (BF_10_ = 46.56; R^2^ = 0.52). Hence, the best model in this statistical framework did not contain structural MRI data. See Table 3 for details on the final model and Supplementary Table 8 for a list of models evaluated and the corresponding BF_10_. The reduced Bayesian regression analysis with only the first component of each imaging method as predictor was in accord with the frequentist approach. The best model identified with BF_10_ criteria was the one with only the posterior ^18^F-AV-1451 and the anterior ^11^C-PK11195 components only as predictors of slope (BF_10_ = 20.81; R^2^ = 0.42), but not the MRI component.

## Discussion

This study demonstrates the independent and combined value of neuroimaging biomarkers for tau pathology (^18^F-AV-1451 PET), neuroinflammation (^11^C-PK11195 PET) and brain atrophy (structural MRI), in predicting longitudinal cognitive decline in patients with Alzheimer’s disease. Baseline markers for tau pathology, neuroinflammation and atrophy in temporo-parietal regions individually predicted cognitive decline, across the spectrum of severity MCI to Alzheimer’s dementia. But, in a multivariable model, cognitive decline was only associated with higher baseline tau pathology in posterior temporo-parietal regions and increased neuroinflammation in the anterior temporal structures. Bayesian analysis confirmed the evidence against the predictive value of MRI atrophy over and above the PET markers of tau pathology and neuroinflammation.

We used PCA to derive the most parsimonious neuroanatomical patterns of pathology that explain most of the imaging variance across the cohort. It is highly efficient for reducing data dimensionality and the problem of multiple comparisons. The PCA indicated two sets of regions (i.e. components) of co-varying tau pathology and neuroinflammation, in anterior versus posterior temporo-parietal regions. We focused on these regions because of their close association with Alzheimer’s disease (Garibotto *et al.*, 2017; Jagust, 2018; Whitwell, 2018), but whole-brain regional data are available on request for other, exploratory, analyses. In patients, the degree of neuroinflammation and tau pathology did not correlate in either anterior or posterior temporo-parietal cortex. Previous studies have considered the *in vivo* association between these two pathological processes in prodromal and early Alzheimer’s disease. Significant associations between tau and neuroinflammation measures have been reported in fronto-temporal regions (Dani *et al.*, 2018), and parahippocampal cortex (Terada *et al*., 2019). Using alternative ligands for tau and inflammation, ^18^F-MK-6240 and ^11^C-PBR28, respectively, positive correlations were found in temporal, parietal and frontal cortex (Zou *et al.*, 2020). However, an earlier study failed to find significant correlations between tau and inflammation (Parbo *et al.*, 2018). Larger sample sizes may be needed to clarify the potential relationship, at different stages of disease.

The participants’ weighting on atrophy, posterior ^18^F-AV-1451 and anterior ^11^C-PK11195 components were separately associated with more rapid cognitive decline (Fig. 4 and Table 2). This result was confirmed by both the one- and two-step univariable prediction approaches. This corroborates the previously reported associations between cognitive deficits in Alzheimer’s disease and the individual effects of tau pathology, neuroinflammation, and downstream cortical atrophy (Femminella *et al.*, 2016; Bejanin *et al.*, 2017; for reviews see Chandra *et al.*, 2019; Melis *et al.*, 2019). Although cross-sectional imaging studies with different PET ligands have reported single associations of cognitive performance with *in vivo* tau (Brier *et al.*, 2016; Cho *et al.*, 2016*b*; Johnson *et al.*, 2016; Ossenkoppele *et al.*, 2016; Pontecorvo *et al.*, 2017; Zou *et al.*, 2020; for a review see Chandra *et al.*, 2019) and microglial activation (Edison *et al.*, 2008; Okello *et al.*, 2009*a*; Fan *et al.*, 2015*a*; Passamonti *et al.*, 2018, 2019; Zou *et al.*, 2020; for a review see Chandra *et al.*, 2019), less is known about their relationship to longitudinal cognitive decline. Previous PET studies in Alzheimer’s dementia and MCI reported that baseline ^18^F-AV-1451 PET uptake correlates with cognitive decline over a period of 6 (Koychev *et al.*, 2017) or 18 months (Pontecorvo *et al.*, 2019). Conversely, microglial activation showed progression over 14–16 months (Fan *et al.*, 2015*b*, 2017), although the predictive value of baseline measures was not reported. Other studies using ^11^C-PBR28 to quantify neuroinflammation over a period of at least 1 year (median 2.7 years) in MCI and Alzheimer’s disease reported increased microglial activation as a function of a significant worsening on the Clinical Dementia Rating scale (Kreisl *et al.*, 2016). Likewise, binding of ^18^F-DPA-714, another TSPO PET ligand, is negatively associated with cognitive performance (Hamelin *et al.*, 2018).

Improving our knowledge of how baseline measures of tau, neuroinflammation, and brain atrophy predict cognitive decline in Alzheimer’s disease may inform future cost-effectiveness of studies in large and epidemiologically representative cohorts of patients. Although other studies have assessed the predictive value of different brain markers on longitudinal cognitive decline in Alzheimer’s disease (for reviews see Chandra *et al.*, 2019; Melis *et al.*, 2019), this study compared the three biomarkers simultaneously (i.e. tau pathology, neuroinflammation, brain atrophy) in patients with amyloid-positive MCI and Alzheimer’s dementia. Our data indicate the added value of PET imaging over and above MRI prognostic markers. Although brain atrophy in isolation is predictive for cognitive decline in Alzheimer’s disease (Jack *et al.*, 2015), when models include tau burden, microglial activation and atrophy jointly, only PET was predictive (Fig. 5 and Table 3). This critical result was confirmed by both frequentist and Bayesian analyses, with evidence against the added value of MRI data on predicting cognitive decline over and above PET assessments. This aligns with cross-sectional studies that report a stronger association of tau molecular imaging than structural MRI with cognitive performance in patients with Alzheimer’s disease (Bejanin *et al.*, 2017; Mattsson *et al.*, 2019). More specifically, in patients with MCI and Alzheimer’s dementia, Benjanin and colleagues (2017) reported an association between regional tau PET binding and cognitive impairment, which was partly mediated by grey matter volumes. Cognition was equally explained by brain atrophy and tau pathology, but after accounting for grey matter values, *in vivo* tau pathology remained correlated with cognitive performance (Bejanin *et al.*, 2017). Likewise, Mattsson *et al*. (2019) found that both ^18^F-AV-1451 PET and structural brain MRI are associated with cognition in Alzheimer’s disease (spanning preclinical, prodromal, and dementia stages), although associations of tau PET indices were stronger than those for MRI markers (Mattsson *et al.*, 2019).

Our data suggest that posterior temporo-parietal ^18^F-AV-1451 binding and anterior temporal ^11^C-PK11195 binding are associated with cognitive decline. In our cohort, they do not interact in their association with cognitive decline, which suggests an additive and independent effect of the two pathological processes on clinical progression, rather than synergy. In patients with Alzheimer’s disease, temporo-parietal cortical tau PET signal is consistent with Braak stage III and above, while in cognitively healthy older individuals, the signal is localized to entorhinal cortex and inferior temporal cortex (Cho *et al.*, 2016*a*; Johnson *et al.*, 2016; for a review see Jagust, 2018). Post-mortem studies have likewise reported tau deposition in the medial temporal cortex in healthy elderly individuals and patients with Alzheimer’s dementia (Jagust, 2018). Tau burden in the entorhinal, limbic, and temporal neocortex relates to cortical atrophy in patients with MCI and Alzheimer’s disease, although not in cognitively normal controls (Timmers *et al.*, 2019). These findings suggest that tauopathy in the medial part of the temporal lobe may be an age-related norm, rather than indicative of Alzheimer’s disease cognitive decline (Femminella *et al.*, 2018). For this reason, tau PET binding here may be a weaker predictor for cognitive decline than tau in the posterior temporo-parietal regions. The co-occurrence with amyloid-β and neuroinflammation may induce the tau spreading from the medial temporal lobe to other cortical regions, which may be associated with downstream neurodegenerative processes and cognitive decline (Mhatre *et al.*, 2015; Jagust, 2018; Perea *et al.*, 2018). This suggests a driving role of neuroinflammation in tau spread and neurodegeneration in Alzheimer’s disease (Yoshiyama *et al.*, 2007; Asai *et al.*, 2015; Maphis *et al.*, 2015), in which activated microglia facilitate tau spread (Maphis *et al.*, 2015; Perea *et al.*, 2018). In addition, it is possible that the relationships between tau, neuroinflammation and cognitive progression are not constant, and that the PET biomarkers would have different prognostic relevance during pre-symptomatic, prodromal and dementia stages of Alzheimer’s disease. Larger studies, or meta-analyses, would be required for adequate power to test such dynamic prognostic models.

There are limitations to our study. TSPO expression in neuroinflammation cascade is complex, and has been found not only in activated microglia but also in other cell types, such as astrocytes and vascular smooth muscle cells (Gui *et al.*, 2019). However, ^11^C-PK11195 is selective for activated microglia over quiescent microglia and reactive astrocytes (Banati, 2002), which favours its utility for imaging activated microglia. In this context, several second-generation PET radioligands for TSPO have been developed since ^11^C-PK11195 (e.g. ^11^C-PBR28 and ^18^F-DPA-714), and used in human studies (Vivash and OBrien, 2016). They are characterized by higher signal-to-noise ratio and lower lipophilicity than ^11^C-PK11195. However, they require genetic analysis to assess a single-nucleotide polymorphism (rs6971), which influences their binding affinity and causes heterogeneity in PET data (Dupont *et al.*, 2017). ^11^C-PK11195 is less affected by this genetic polymorphism, especially between high and mixed affinity binders (Guo *et al.*, 2012; Kobayashi *et al.*, 2018) that represent ∼90% of the Caucasian population (Owen *et al.*, 2012), although a small difference in ^11^C-PK11195 binding in the CNS remains a possibility (Fujita *et al.*, 2017). Second, the cross-sectional nature of our imaging assessment does not enable a mediation analysis, or support inferences on the direction of causality between tau pathology, microglial activation progression and cognitive decline. However, both processes predict the rate of cognitive deterioration in Alzheimer’s disease. Third, the modest sample size of our cohort limited the applicability of the one-step prediction procedure with multiple predictors, which may lead to a more precise prediction than the two-step procedures. For the multivariable regression model the sample size was reduced to *n *=* *26 because of the exclusion of controls (who underwent ^18^F-AV-1451 or ^11^C-PK11195 PET, but not both, to limit radiation exposure). However, both frequentist and Bayesian multivariable approaches give similar results, aligning with those obtained by the one-step prediction. The convergence between the statistical models (i.e. LGCM with predictors, linear regression and Bayesian model) mitigates against sample-dependant biases on the estimation of the most parsimonious model. The replication of these findings with larger and multicentre clinical cohorts will represent an important next step to establish the replicability and generalizability of our results. Fourth, the interval between cognitive assessment and imaging varied. However, we sought to mitigate this confound by including the interval in the statistical analyses, and note that the intervals were small compared with the 3-year follow up.

We conclude that PET markers of regional pathological processes are stronger predictors than atrophy, as measured by MRI, of clinical progression in patients with symptomatic Alzheimer’s disease. The predictive models were convergent in identifying tau burden in posterior cortical regions and neuroinflammation in the anterior temporal lobe as imaging predictors of cognitive decline in the clinical spectrum of Alzheimer’s disease. In contrast, atrophy predicted cognitive decline only if considered individually but not over and above the effects of tau burden and inflammation. Our findings support the use of PET imaging of tau pathology and microglial activation for prognostication and patients’ stratification in clinical trials.

## Supplementary Material

awaa088_Supplementary_Data

## Abbreviations

ACE-R =: revised Addenbrooke’s Cognitive Examination
BP_ND_ =: non-displaceable binding potential
CFI =: comparative fit index
LGCM =: latent growth curve model
MCI =: mild cognitive impairment
RMSEA =: root-mean-square error of approximation
SRMR =: standardized root mean-square residual
