# Supplementary material for: Microglial activation and tau burden predict cognitive decline in Alzheimer’s disease
Source: Brain. 2020 May 7;143(5):1588–602. doi: 10.1093/brain/awaa088 (PMC7241955; doi:10.1093/brain/awaa088)
Supplement: awaa088_Supplementary_Data [file awaa088_supplementary_data.pdf]

Supplementary Material

Supplementary Figure 1. Schematic representation of statistical analyses for one-step and two-step prediction procedures. Abbreviation: ACE-R: Addenbroke's Cognitive Examination – Revised; i: intercept; s: slope; PC: principal component; AV: [^{18}F]AV-1451; PK: [^{11}C]PK11195; Bas: baseline; y: years.

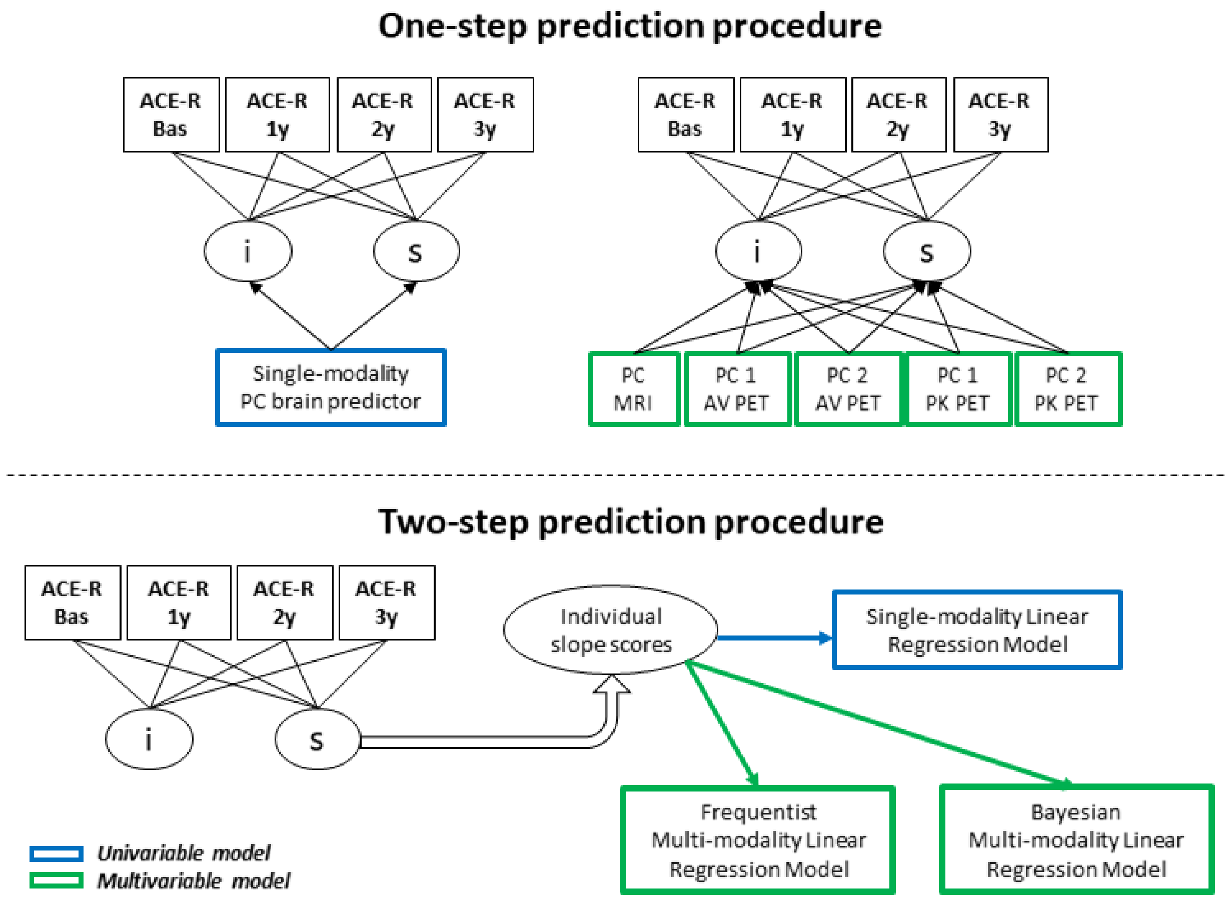

Supplementary Table 1. Counts and group comparisons between controls and patients who underwent ligand-specific PET scans on a GE Advance PET scanner (GE Healthcare, Waukesha, USA) and a GE Discovery 690 PET/CT. Chi-square tests resulted not significant for both tracers, indicating that subgroups of controls on different scanners for each ligand-specific PET were balanced with those of patients. In addition, to minimise differences in the data, the acquisition protocols (injected activity, scan duration) and image reconstruction strategies (frame durations, reconstruction algorithm) were matched between scanners. The transaxial reconstructed field of view (30 cm) and voxel dimension (2.34 mm) were invariant across scanners, as was the data analysis methodology. The use of scanners from the same manufacturer was also beneficial regarding the data corrections applied during image reconstruction.

[18F]AV1451 PET				
		**scanner PET**		**Total**
		**ADVANCE**	**DISCOVERY**	
**Group**	**AD/MCI**	18	8	26
	**Controls**	8	6	14
**Total**		26	14	40
**Group comparison**		$\chi2(1)=0.58$; $p=0.45$		
[11C]PK11195 PET				
		**scanner PET**		**Total**
		**ADVANCE**	**DISCOVERY**	
**Group**	**AD/MCI**	18	8	26
	**Controls**	13	2	15
**Total**		31	10	41
**Group comparison**		$\chi2(1)=1.57$; $p=0.21$		

Supplementary Table 2. The fifteen Alzheimer's disease related cortical regions of interest, considered for our principal component analyses. The name of each region is reported in the first column; while the second column gives the corresponding region numbers in the Hammers atlas.

**Region**	**Numbers (R/L)**
Hippocampus	1/2
Amygdala	3/4
Anterior medial temporal lobe	5/6
Anterior lateral temporal lobe	7/8
Parahippocampal gyri	9/10
Superior posterior temporal gyrus	11/12
Middle and inferior temporal gyrus	13/14
Fusiform gyrus	15/16
Insula	21/20
Posterior cingulate gyrus	27/26
Posterior temporal lobe	31/30
Inferiolateral parietal lobe	33/32
Superior parietal gyrus	63/62
Cuneus	67/66
Superior anterior temporal gyrus	83/82

Supplementary Table 3. Demographic and clinical characteristics for the subsample of control and patients groups.

	**Controls AV PET**	**Control PK PET**	**Group difference**	**MCI+**	**AD**	**Group difference**
**N**	**14**	15		**14**	**12**	
**Disease Duration (years - mean ± SD)**	-	-	-	3.8±2.3	3.4±1.8	t(24)=0.60; p=0.558
**Sex (Female/Male)**	7/7	8/7	$\chi 2$(1)=0.03; p=0.858	7/7	5/7	$\chi 2$(1)=0.18; p=0.671
**Age (years - mean ± SD)**	66.9±7.6	69.6±6.8	t(27)=-1.04; p=0.308	74.6±6.4	69.1±10.4	t(24)=1.64; p=0.115
**Education (years - mean ± SD)**	15.8±1.9	14.1±3.0	t(27)=1.77; p=0.09	12.3±2.8	14.0±3.5	t(24)=-1.39; p=0.176
**ACE-R Baseline (mean ± SD)**	95.4±3.1	95.5±4.5	t(27)=1.26; p=0.220	80.6±6.5	74.5±10.8	t(17.4)=1.70; p=0.107

Abbreviations AV: [^{18}F]AV-1451; PK: [^{11}C]PK11195; ACE-R: Addenbroke's Cognitive Examination – Revised; SD: standard deviation; t(): t-test; p: p-value

Supplementary Table 4. Regional weights of the structural MRI component (left), and rotated regional weights of [^{18}F]AV-1451 components (middle) and the [^{11}C]PK11195 components (right). Abbreviations: GM=Grey Matter; Comp=Component.

**GM volumes**		**[^{18}F]AV-1451**			**[^{11}C]PK11195**		
**Region**	**Comp 1**	**Region**	**Comp 1**	**Comp 2**	**Region**	**Comp 1**	**Comp 2**
Posterior temporal lobe	0.910	Posterior cingulate gyrus	0.923	0.338	Anterior lateral temporal lobe	0.920	0.201
Middle and inferior temporal gyrus	0.909	Superior parietal gyrus	0.915	0.363	Parahippocampal gyri	0.906	-0.055
Parahippocampal gyri	0.903	Cuneus	0.872	0.380	Anterior medial temporal lobe	0.880	0.078
Amygdala	0.901	Inferiolateral parietal lobe	0.872	0.451	Hippocampus	0.835	0.057
Anterior medial temporal lobe	0.891	Posterior_temporal_lobe	0.791	0.587	Fusiform gyrus	0.796	0.187
Fusiform gyrus	0.884	Parahippocampal gyri	0.725	0.483	Superior anterior temporal gyrus	0.743	0.389
Superior posterior temporal gyrus	0.879	Fusiform gyrus	0.660	0.643	Amygdala	0.723	0.281
Anterior lateral temporal lobe	0.875	Hippocampus	0.274	0.891	Middle and inferior temporal gyrus	0.680	0.626
Hippocampus	0.869	Anterior medial temporal lobe	0.387	0.876	Superior parietal gyrus	0.282	0.879
Posterior cingulate gyrus	0.863	Superior anterior temporal gyrus	0.395	0.858	Posterior cingulate gyrus	-0.340	0.847
Inferiolateral parietal lobe	0.840	Insula	0.460	0.845	Inferiolateral parietal lobe	0.434	0.831
Superior parietal gyrus	0.821	Amygdala	0.508	0.791	Superior posterior temporal gyrus	0.438	0.804
Superior anterior temporal gyrus	0.812	Anterior lateral temporal lobe	0.530	0.768	Posterior temporal lobe	0.513	0.794
Insula	0.782	Middle and inferior temporal gyrus	0.687	0.694	Insula	0.385	0.755
Cuneus	0.735	Superior posterior temporal gyrus	0.662	0.688	Cuneus	-0.147	0.563

Supplementary Table 5. Results for the univariable regression models on slope (left) and intercept (right) across all population with age, sex and education as covariates.

		**Dependent variable: cognitive slope**					**Dependent variable: cognitive intercept**				
**Model**		**Est**	**Std Error**	**Std Beta**	**t value**	**p**	**Est**	**Std Error**	**Std Beta**	**t value**	**p**
**MRI (N=55)**	**(Intercept)**	-13.81	5.99		-2.31	0.025	55.06	10.59		5.20	<0.001
	**MRI comp**	4.03	0.65	0.70	6.19	**<0.001**	7.07	1.15	0.64	6.14	**<0.001**
	**Age**	0.18	0.07	0.27	2.45	0.018	0.25	0.13	0.20	1.97	0.055
	**Sex**	0.82	1.14	0.08	0.72	0.472	2.05	2.01	0.10	1.02	0.314
	**Education**	-0.13	0.20	-0.08	-0.67	0.505	0.92	0.35	0.27	2.59	0.012
**AV 1 (N=40)**	**(Intercept)**	-7.57	8.18		-0.93	0.361	67.95	11.94		5.69	<0.001
	**AV comp 1**	-3.67	0.81	-0.64	-4.56	**<0.001**	-7.19	1.17	-0.67	-6.12	**<0.001**
	**Age**	-0.04	0.09	-0.06	-0.39	0.699	-0.20	0.14	-0.16	-1.45	0.156
	**Sex**	0.97	1.54	0.09	0.63	0.533	3.77	2.24	0.18	1.68	0.102
	**Education**	0.38	0.26	0.21	1.49	0.144	2.00	0.37	0.57	5.33	<0.001
**AV 2 (N=40)**	**(Intercept)**	-18.95	9.18		-2.06	0.047	47.54	16.38		2.90	0.006
	**AV comp 2**	-2.26	0.90	-0.39	-2.51	**0.017**	-1.52	1.61	-0.14	-0.95	**0.351**
	**Age**	0.14	0.10	0.21	1.34	0.190	0.12	0.18	0.09	0.63	0.532
	**Sex**	1.55	1.82	0.14	0.85	0.402	3.68	3.25	0.17	1.13	0.265
	**Education**	0.30	0.30	0.16	1.02	0.316	1.88	0.53	0.54	3.53	0.001
**PK 1 (N=41)**	**(Intercept)**	-4.72	9.55		-0.49	0.624	71.26	16.81		4.24	<0.001
	**PK comp 1**	-2.65	0.93	-0.46	-2.86	**0.007**	-4.37	1.63	-0.42	-2.68	**0.011**
	**Age**	0.04	0.11	0.05	0.32	0.751	0.00	0.20	0.00	-0.01	0.995
	**Sex**	0.37	1.71	0.03	0.22	0.830	0.45	3.00	0.02	0.15	0.882
	**Education**	-0.16	0.28	-0.09	-0.59	0.561	0.90	0.49	0.27	1.83	0.076
**PK 2 (N=41)**	**(Intercept)**	-17.55	8.85		-1.98	0.055	51.04	16.37		3.12	0.004
	**PK comp 2**	-2.50	1.00	-0.41	-2.50	**0.017**	-2.17	1.85	-0.20	-1.17	**0.248**
	**Age**	0.13	0.11	0.18	1.21	0.232	0.18	0.20	0.14	0.92	0.362
	**Sex**	0.92	1.73	0.08	0.53	0.598	1.49	3.19	0.07	0.47	0.643
	**Education**	0.27	0.30	0.15	0.89	0.378	1.39	0.56	0.41	2.46	0.019

Abbreviations AV: [^{18}F]AV-1451; PK: [^{11}C]PK11195; Std: standard; p=uncorrected p-values

Supplementary Table 6. Results for the univariable regression models on cognitive slope in patients.

Model		Estimate	Std Error	Std Beta	t value	p	Adj R^2 (std err)	F	p
**MRI (N=26)**	**(Intercept)**	-5.02	1.25		-4.00	.001	0.167 (5.18)	6.02	0.022
	**MRI component**	3.08	1.26	0.45	2.45	.022			
**AV 1 (N=26)**	**(Intercept)**	-6.23	0.97		-6.43	.000	0.271 (4.84)	10.29	0.004
	**AV component 1**	-2.63	0.82	-0.55	-3.21	.004			
**AV 2 (N=26)**	**(Intercept)**	-6.56	1.13		-5.78	.000	0.007 (5.65)	1.18	0.289
	**AV component 2**	-1.08	0.99	-0.22	-1.09	.289			
**PK 1 (N=26)**	**(Intercept)**	-6.36	1.06		-6.01	.000	0.131 (5.29)	4.78	0.039
	**PK component 1**	-2.02	0.92	-0.41	-2.19	.039			
**PK 2 (N=26)**	**(Intercept)**	-6.54	1.06		-6.20	.000	0.116 (5.34)	4.27	0.05
	**PK component 2**	-2.11	1.02	-0.39	-2.07	.050			

Abbreviations AV: [^{18}F]AV-1451; PK: [^{11}C]PK11195; Std: standard; Adj: adjusted, p=uncorrected p-values

Supplementary Table 7. Results for the multiple regression model in patients with Alzheimer's dementia and Mild Cognitive Impairment. For each of the three multiple regression models applied and described in the methods' section, the estimated coefficients are reported for the full/initial model and the final model, indicated by the stepwise backward elimination.

Dependent variable	Model		Estimate	Std Error	Std Beta	t value	P	Adj R^2 (std err)	F	p
slope	Full	(Intercept)	-7.09	11.14		-0.64	0.533	0.389 (4.43)	2.99	0.027
		MRI component	0.85	1.39	0.12	0.62	0.547			
		AV component 1	-2.30	1.07	-0.48	-2.14	0.047*			
		AV component 2	-1.18	0.94	-0.24	-1.26	0.224			
		PK component 1	-1.59	1.10	-0.32	-1.45	0.164			
		PK component 2	-1.28	1.10	-0.24	-1.17	0.260			
		Age	0.00	0.15	0.01	0.03	0.980			
		Education	0.11	0.36	0.06	0.30	0.765			
		Sex	0.85	2.02	0.08	0.42	0.678			
	Full (only brain measures)	(Intercept)	-5.03	1.01		-4.99	0.000*	0.474 (4.12)	5.50	0.002
		MRI component	0.84	1.22	0.12	0.67	0.501			
		AV component 1	-2.20	0.74	-0.46	-2.96	0.008*			
		AV component 2	-1.07	0.82	-0.21	-1.31	0.205			
		PK component 1	-1.68	0.86	-0.39	-1.95	0.066			
		PK component 2	-1.25	0.87	-0.23	-1.43	0.168			
	Final	(Intercept)	-5.41	0.87		-6.19	0.000*	0.458 (4.18)	8.05	0.001
		AV component 1	-2.57	0.71	-0.54	-3.60	0.002*			
		AV component 2	-1.64	0.74	-0.33	-2.21	0.038*			
		PK component 1	-1.92	0.74	-0.39	-2.59	0.017*			

Abbreviations AV: [^{18}F]AV-1451; PK: [^{11}C]PK11195; Std: standard; Adj: adjusted
*Significance code: *= p<0.05*

Supplementary Table 8. Model comparison on the Bayesian multiple regression with cognitive slope as the dependent variable and brain imaging components and demographic variables as predictors. The models are ordered by the higher Bayes Factor (BF_{10}) to the lowest. The table shows the ten most likely models, the null model, and the least likely model at the bottom. The Bayes Factor has been calculated for each model to the null model with respect to the null model. By convention, a BF>3 indicates positive evidence in favour of the alternate model, BF>10 strong evidence and BF>100 very strong evidence (Kass and Raftery, 1995). The top three ranked models are therefore significantly 'better' prognostic models than any other model, including any model with MRI.

Models	P(M)	P(M|data)	BF_M	BF_{10}	R^2
AV COMP1 + AV COMP2 + PK COMP1	0.004	0.033	8.744	46.560	0.523
AV COMP1 + PK COMP1 + PK COMP2	0.004	0.032	8.333	44.443	0.521
AV COMP1 + AV COMP2 + PK COMP1 + PK COMP2	0.004	0.030	7.804	41.705	0.569
MRI COMP + AV COMP1 + PK COMP1 + PK COMP2	0.004	0.018	4.680	25.31	0.543
MRI COMP + AV COMP1 + AV COMP2 + PK COMP1	0.004	0.016	4.112	22.289	0.536
MRI COMP + AV COMP1 + PK COMP2	0.004	0.016	4.031	21.856	0.481
AV COMP1 + PK COMP1	0.004	0.015	3.835	20.806	0.417
MRI COMP + AV COMP1	0.004	0.015	3.817	20.714	0.417
AV COMP1 + AV COMP2 + PK COMP1 + Sex	0.004	0.015	3.808	20.663	0.532
MRI COMP + AV COMP1 + AV COMP2 + PK COMP1 + PK COMP2	0.004	0.014	3.722	20.205	0.579
…					
Null model	0.004	7.120e -4	0.182	1	0
…					
AV COMP2 + Education + Sex	0.004	1.398e -4	0.036	0.196	0.082

Abbreviations AV: [^{18}F]AV-1451; PK: [^{11}C]PK11195; COMP: component; BF: Bayes factor

Supplementary material and methods

Statistical analyses

Latent Growth Curve Model with quadratic term for cognitive decline. Hypothesising that the cognitive decline over 3 years follows a linear trajectory in aging and Alzheimer's disease, we compared the linear model of change with a quadratic model. To determine whether a quadratic function is appropriate, the models with and without the quadratic effect were compared with a likelihood ratio test.

Two-step frequentist prediction procedure on patients only. We applied a two-step prediction procedure to patient data: 1) extracting individual slope values from the initial LGCM for cognitive data across the 26 patients, and 2) including these values as dependent variable in linear regression models with brain imaging components as predictors. We present both frequentist and Bayesian analyses to ensure inferential robustness and allowing us to quantify evidence in favour of the null hypothesis.

Supplementary results

Latent Growth Curve Model with the quadratic term. The LGCM with the quadratic term on longitudinal ACE-R scores fitted the data adequately ($\chi2(4)=2.78$, p=0.595; RMSEA=0.00 [0.00 – 0.21], CFI=1.00, SRMR=0.014). However, comparing the model fit between the linear and the quadratic LGCM on ACE-R scores, the ANOVA test (anova() R function) did not find significant differences (Chisq Diff= 3.04, p=0.219) between the linear model (AIC=1249.2) and the quadratic one (AIC=1250.3). This means that adding the quadratic term does not improve the model of cognitive decline.

Two-step frequentist prediction procedure for intercept. Across all subjects, the initial cognitive performance (intercept in LGCM on ACER scores) was significantly associated with: 1) the MRI component weighting (Std Beta=0.67, p<0.001); 2) the posterior [^{18}F]AV-1451 (Std Beta=-0.55, p<0.001); 3) and anterior [^{11}C]PK11195 (Std Beta=-0.46, p=0.003), surviving Bonferroni's correction. Non-significant correlations of slope were found with the anterior [^{18}F]AV-1451 (Std Beta=-0.15, p=0.35), and the posterior [^{11}C]PK11195 (Std Beta=-0.06 p=0.72). These results remained unchanged if including age, sex and education as covariates in the models (Supplementary Table 5). In patients, the final model of multiple regression on cognitive intercept (adjusted R^2 = 0.519, Std Error= 5.81; p<0.001) included the education variable (Est=1.02, Std Error=0.38, p=0.014), the MRI component (Est=3.61, Std Error=1.46, p=0.021) and the posterior [^{18}F]AV1451 (Est=-3.99, Std Error=1.05, p=0.001) as predictors.

Two-step frequentist prediction procedure on patients only. The cognitive LGCM on patients converged using robust maximum likelihood estimation and yielded adequate fit ($\chi2(8)=9.40$, p=0.310; RMSEA=0.08 [0.00 – 0.26], CFI=0.99, SRMR=0.07). The mean of the intercept was 77.52 (SE=1.73, z-value=44.93, Std Est =9.27, p<0.001) and average cognition declined over time (slope, estimate (est)=-6.87, SE=1.35, z-value=-5.09, Std Est=-1.09, p<0.001).

Across all patients, the rate of cognitive decline was associated with: 1) the MRI component (Std Beta=0.45, p=0.034); 2) the posterior [18F]AV-1451 (Std Beta=-0.53, p=0.005); 3) anterior [^{11}C]PK11195 (Std Beta=-0.40, p=0.041); and the posterior [^{11}C]PK11195 (Std Beta=-0.40, p=0.05). Running the regression model with all imaging components and demographic variables, the final model on cognitive slope selected by the backward selection (adjusted R^2 = 0.437, Std Error= 7.46; p=0.001) included both [^{18}F]AV-1451 components (#1: Est=-2.64, Std Error=0.77, p=0.002; #2: Est=-1.77, Std Error=0.81, p=0.039), and the anterior [^{11}C]PK11195 (#1: Est=-2.03,

Std Error=0.80, p=0.019) as predictors. We estimated the comparable models using Bayesian regression. The results were in accord with the backward selection method, with the best model including both [18F]AV-1451 components and the anterior [^{11}C]PK11195 component (BF10 = 32.85; R^2 = 0.504).
